# Unified approach to growth and aging in biological, technical and biotechnical systems

**DOI:** 10.1186/2193-1801-1-7

**Published:** 2012-07-09

**Authors:** Paolo Castorina, Philippe Blanchard

**Affiliations:** 1 Fakultät für Physik, Universität Bielefeld, D-33501 Bielefeld, Germany; 2 Dipartimento di Fisica, Universita’ di Catania, 95100 Catania, Via Santa Sofia 64, Italy; 3 INFN, Sezione di Catania, 95100 Catania, Italy

**Keywords:** Growth laws, Aging, Tumor growth, 89.75.-k

## Abstract

Complex systems, in many different scientific sectors, show coarse-grain properties with simple growth laws with respect to fundamental microscopic algorithms. We propose a classification scheme of growth laws which includes human aging, tumor (and/or tissue) growth, logistic and generalized logistic growth and the aging of technical devices. The proposed classification permits to evaluate the aging/failure of combined new bio-technical “manufactured products”, where part of the system evolves in time according to biological-mortality laws and part according to technical device behaviors. Moreover it suggests a direct relation between the mortality leveling-off for humans and technical devices and the observed small cure probability for large tumors.

## Background

Complex systems with millions of interacting elementary parts are often considered computationally irreducible Wolfram ( [1984]); Wolfram ( [2002]) which means that the only way to decide about their evolution is to let them evolve in time.

On the other hand, there is an impressive number of experimental verifications, in many different scientific sectors, that coarse-grain properties of systems, with simple laws with respect to fundamental microscopic alghoritms, emerge at different levels of magnification providing important tools for explaining and predicting new phenomena.

In this respect, a priori unrelated systems show similar emergent properties and if an unexpected effect is found experimentally in a field, a similar effect, “mutatis mutandis”, should also be sought in similar experiments in other fields. Therefore a useful tool to greatly facilitate the cross fertilization among different fields of research is a general classification of growth laws Castorina et al. ( [2006]).

A very important example is the Gompertz law (GL) Gompertz ( [1825]) which applies to human mortality tables (i.e. aging) and tumor growth Steel ( [1977]); Wheldon ( [1988]); Norton ( [1988]).

In general, a growth problem is characterized by a function *f* (*t*), which describes the time evolution of some macroscopic quantity, and by the specific rate, *α*, defined as (1/*f*)(*df*/*dt*)=*α*(*t*). In the GL *α*has an exponential dependence on time: 1

where *a* and *b* are constants. In aging *f* (*t*) indicates the survival probability; while with regards to tumor growth it corresponds to the number of cells *N*(*t*) (depending on the specific case *a* and *b* can be positive or negative).

For technical devices the specific rate of the survival probability has a power-law time behavior 2

with *n*>1, called Weibull law (WL) Barlow and Proschan ( [1975]); Rigdon and Basu ( [2000]). The analogy with the biological systems is intriguing (for clarity, as necessary, one defines the specific rate *α*_*h*_(*t*) for the human mortality, *α*_*f*_(*t*) for the technical systems and *α*_*c*_(*t*) for tumor growth) and deeper than the similarity between eq. (1) and eq. (2).

Indeed, many independent analyses of experimental data on humans and animals suggest that at advanced ages (more than 85-90 years for humans) there is a deceleration in mortality Gavrilov and Gavrilova ( [1991]); Vaupel et al. ( [1998]); Olshansky ( [1998]): in the large range 20 - 85 years for humans the mortality rate is well described by the Gompertz law and then there is a late-life mortality (although a definite conclusion has yet to be reached Gavrilov and Gavrilova ( [2011])). A similar trend is observed for technical devices Economos ( [1979]), confirming the analogy between biological and technical systems.

The understanding of aging and of late-life mortality is still an open problem and many interesting models have been proposed to explain the similar behavior in metabolic systems and in technical devices Gavrilov and Gavrilova ( [2001]). Moreover, a unifying language for the description of performance of metabolic and technical production and distribution has been recently suggested Becker et al. ( [2011]) to implement the idea that the robustness of metabolic systems with respect to enviromental changes could represent a useful model for technical systems.

In this letter, rather than focusing on specific models, we shall address the generalization of the classification scheme of growth laws to include human aging, tumor (and/or tissue) growth, logistic and generalized logistic growth and the aging of technical devices. We shall consider two applications of the proposed approach: a) a method to evaluate the aging/failure of combined new bio-technical “manufactured product”, where part of the system evolves in time according to biological-mortality laws and part is a technical device; b) an interpretation of the “tumor size effect”, i.e. the small cure probability for large tumor Stanley et al. ( [1977]); Bentzen and Thomas ( [1996]); Huchet et al. ( [2003]), in analogy with the late-life mortality in aging.

## Results

Let us start with the general classification scheme. It turns out that a classification of the growth laws according to the simple equation (1/*f*)(*df*/*dt*)=*α*(*t*) is obtained by considering the power expansion in *α*of the function ( see ref. Castorina et al. ( [2006]) for details) 3

which for *b*_0_=0 and *b*_*i*_=0 for *i*>1 gives a time independent specific rate *α*_0_ and therefore an exponential growth; for *b*_0_≠0 and *b*_*i*_=0 for *i*>1 describes a linear time dependent specific rate and again an exponential growth; at the first order in *α*, for *b*_0_=0, *b*_1_≠0 and *b*_*i*_=0 for *i*>1, reproduces an exponential time behavior of the specific growth and therefore the GL; the second order term , *O*(*α*^2^), for *b*_0_=0, *b*_1_*b*_2_≠0 and *b*_*i*_=0 for *i*>2 generates the logistic and generalized logistic growth.

The feedback effect, that is the dependence of the specific growth rate *α* on the function *f* (*t*), can be easily derived by the temporal behaviour of the specific rate. For the GL for a growing number of cells, *N*(*t*), one has the well known logarithmic non linearity, 4

and for the (generalized) logistic law one gets the typical power-law behavior 5

where *a*,*b*,*c*,*γ* are constants and the carrying capacity,  ,corresponds to *α*=0.

In order to describe technical devices, the previous classification scheme has to be generalized since the specific growth rate of Weibull law has a power law dependence on time which is not reproduced by eq. (3). The behavior ,with *n* positive integer, corresponds to terms O( *α*^(*n*−1)/*n*^) in the expansion of *Φ*(*α*) and therefore for a general classification scheme of the specific growth/aging/failure rate of biological and technical systems one has to consider: 6

Note that: a) 0<(*n*−1)/*n*<1 and the nth term in the power series in *α*^(*n*−1)/*n*^tends for large *n* to *α*, i.e. to the Gompertz law; b) the term *b*_0_≠0, i.e. the exponential growth, has been neglected because one considers the GL, the generalized logistic or more complex growth laws for the biological systems (there is no problem to include this term in the expansion) ; 3) the first sum in the expansion has fractional powers that recall a Puiseux expansion.

As a by-product of the proposed classification scheme one can easily evaluate the aging/failure of combined new bio-technical “manufactured products” by taking explicitely into account the mutual “interference” between the aging behavior of the biological part and the failure of the technical one. The “interference” effect strongly depends on the typical time scales in the coefficients *c*_*n*_ and *b*_*n*_ in the previous expansion: if the life-time of the technical device is much larger than the life-time of the biological part ( or viceversa) there is essentially no effect Muller et al. ( [1988]).

Let us first consider aging/failure of a combined bio-technological “manufactured product”, where part of the system evolves in time according to GL, i.e. the term *O*(*α*), and the behavior of technical part is described by a single term *O*(^*αn*−1/*n*^),i.e. 7

By introducing dimensionless variables in time unit 1/*b*_1_, i.e. *τ*=*b*_1_*t*,  and , after simple calculations the time dependence of the specific rate is given by: 8

where . Of course in the limit  one recovers the GL and for  the Weibull one. By previous equation, for , one obtains: 9

which describes the combined effect of the two growth laws. The quantitative effect is depicted in Figures ( 1, 2) where the previous function is plotted for different values of *n* at fixed  and for various values of  at fixed *n*.

**Figure 1:**
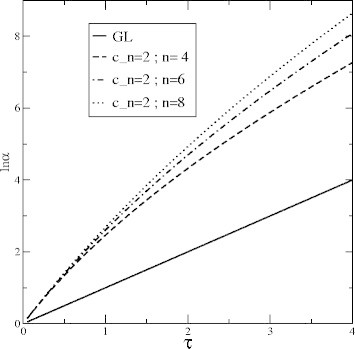
**Comparison of the GL, the WL and the combined effect for a biotechnical device for**.*τ*=*b*_1_*t* and the curves are for a fixed value of the coefficient  and different values of *n*=4,6,8

**Figure 2:**
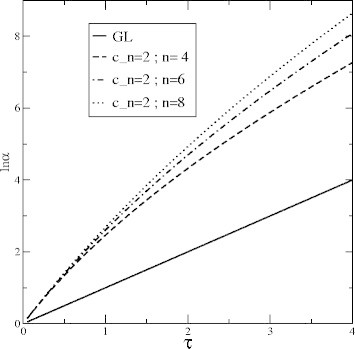
**Comparison of the GL, the WL and the combined effect for a biotechnical device for**. *τ*=*b*_1_*t* and the curves are for *n*=6 and the coefficient

The next step is to include the term  in the expansion of *Φ*(*α*) (*b*_2_ is dimensionless) which corresponds to a generalized logistic evolution. As we shall see this term is crucial in understanding the late-life mortality effect.

By repeating analogous calculations it turns out that 10

**Figure 3:**
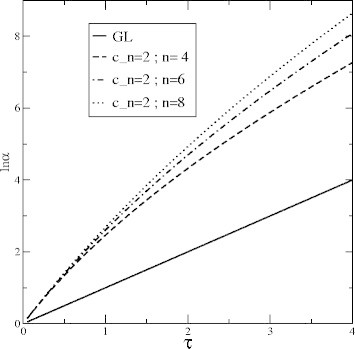
**Comparison for** of the GL, the WL and the effects of ***O*** (*α*^2^) term for ***n=6***,  and ***b***_***2***_**=− 0.02**

In Figure 3 is shown that the term  completely changes the time evolution with respect to GL and/or WL producing a leveling-off of the specific rate.

Therefore the general expansion of *Φ*(*α*) in eq. (6) can describe the aging/failure of any biological and technical system including the leveling-off at late mortality which is obtained by taking into account the term *O*(*α*^2^) in *Φ*(*α*), i.e. by the transition from the GL or WL to a logistic type law Horiuchi and Wilmoth ( [1998]).

The proposed unification scheme suggests a practical method to understand growth patterns. Given a set of data on some growth process, the first step of the analysis is a fit in power of *α* of the derivative of the specific growth rate, i.e. of the function *Φ*(*α*). Therefore : a) if the best fit is linear, the growth is a Gompertzian one; b) if the best fit is quadratic, look at the sign of the coefficients of the expansion. For *b*_1_>0 and *b*_2_<0 the growth is logistic (or generalized logistic) corresponding to a competitive dynamics; c) if the best fit indicates a fractional power the growth follows the WL. Of course, it is always possible to obtain a better agreement with data by increasing the number of coefficients. However, should increasing the number of parameters indicate only a marginal improvement in the description of data one concludes that the added terms in the expansion are irrelevant.

## Discussion and conclusions

Let us now consider the cross-fertilization among different sectors.

As previously discussed, there is a deceleration of mortality in aging at late time which is described as a “transition” from a Gompertz law to a generalized logistic behavior. On the other hand, tumors evolve in time according to the GL. The obvious indications is to verify if a phenomenon corresponding to the deceleration of mortality, i.e. a transition from the GL to a power law, exists for cancer growth at a later time. As we shall see, this aspect has strong consequences on the therapy.

For tumor growth the *b*_1_*α* term gives the GL in eq. (4) and the introduction of the *O*(*α*^2^) term corresponds to the power law non-linear feedback in eq. (5). Therefore one has to investigate if at late-life of a tumor growth there is such a modification in the dependence of the specific growth rate on the cell number *N*(*t*). Since direct informations “in vivo” are almost impossible, the question has to be addressed in an indirect way by considering radiotherapy.

The radiotherapic tumor treatment consists in series of radiation doses at fixed time intervals. However tumors start to re-grow in the interval between two treatments : the re-growth during radiotherapy is therefore an important clinical parameter Kim and Tannock ( [2005]) and the probability of treatment benefit critically depends on the tumor re-growth pattern.

The so called “tumor size effect” is a reduction of radiotherapeutic results for large tumors ( which , presumably, has grown since long time). The dependence of the surviving fraction on the tumor volume was already observed by Stanley et al. in 1977 in lung tumors Stanley et al. ( [1977]) and re-emphasized by Bentzen et al. and Huchet et al. in Bentzen and Thomas ( [1996]); Huchet et al. ( [2003]).

The effect of re-growth rate on radiotherapy has been quantitatively investigated in ref. Castorina et al. ( [2007]) and the results clearly indicate that to understand the tumor size effect the re-growth rate for large tumor has to follow a power law Guiot et al. ( [2003]) rather than the GL.

From this point of view the “tumor size effect” is a phenomenon which indicates that in late -time tumor growth there is a change from a GL specific rate to a power law behavior, corresponding to the deceleration in mortality at advanced age.

One should conclude that such a common feature in aging and in failure in biological and/or technical systems should be considered as a “bifurcation” or a “phase transition” in the specific growth rate at large time from GL or WL to a logistic or generalized logistic behavior.

In closing, the general expansion of *Φ*(*α*) in eq. (6) can describe the growth/aging/failure of biological and technical systems and the transition to a different (“phase”) specific growth rate at late-life could be a common feature of those systems independently on the microscopic dynamics.
